# Organic Acids and Potential for Modifying the Avian Gastrointestinal Tract and Reducing Pathogens and Disease

**DOI:** 10.3389/fvets.2018.00216

**Published:** 2018-09-06

**Authors:** Dana K. Dittoe, Steven C. Ricke, Aaron S. Kiess

**Affiliations:** ^1^Department of Food Science and Center for Food Safety, University of Arkansas, Fayetteville, AR, United States; ^2^Department of Poultry Science, Mississippi State University, Starkville, MS, United States

**Keywords:** poultry, organic acids, lactic acid producing bacteria, prebiotics, microbiome

## Abstract

Recently, antibiotics have been withdrawn from some poultry diets; leaving the birds at risk for increased incidence of dysbacteriosis and disease. Furthermore, mortalities occurring from disease contribute between 10 to 20% of production cost in developed countries. Currently, numerous feed supplements are being proposed as effective antibiotic alternatives in poultry diets, such as prebiotics, probiotics, acidic compounds, competitive exclusion products, herbs, essential oils, and bacteriophages. However, acidic compounds consisting of organic acids show promise as antibiotic alternatives. Organic acids have demonstrated the capability to enhance poultry performance by altering the pH of the gastrointestinal tract (GIT) and consequently changing the composition of the microbiome. In addition, organic acids, by altering the composition of the microbiome, protect poultry from pH-sensitive pathogens. Protection is further provided to poultry by the ability of organic acids to potentially enhance the morphology and physiology of the GIT and the immune system. Thus, the objective of the current review is to provide an understanding of the effects organic acids have on the microbiome of poultry and the effect those changes have on the prevalence of pathogens and diseases in poultry. From data reviewed, it can be concluded that the efficacy of organic acids on shifting microbiome composition is limited to the time of administration, the composition of the organic acid product, and the current health conditions of poultry.

## Introduction

With the removal of antibiotics from some poultry integrators and the implementation of antibiotic-free birds (ABF), the industry is challenged with identifying a valid alternative to antibiotics with similar capabilities to that of antibiotics (1, 2). As antibiotics have been noted to improve weight gain, through the reduction of subclinical and clinical infection by mitigating the presence of bacteria in the gastrointestinal tract (GIT) and consequently reducing nutrient competition, immune stimulation, thinning the intestinal wall, and enhancing nutrient digestibility (3, 4), these are considered the qualities expected of an effective alternative. Many antibiotic alternative products improve growth performance characteristics of poultry by directly impacting the environment of the GIT, such as altering the bacterial populations, physiology, and the pH of the GIT (5). Although there are numerous alternatives currently on the market, organic acids are a valid alternative with the capability to reduce pathogenic bacteria and increase nutrient digestibility through their effect on the pH in the GIT (5–7). Because the digestive process extensively includes microbial fermentation, organic acids are commonly produced by beneficial bacteria (probiotics) present in the crop, intestines, and ceca (3). Furthermore, the supplementation of prebiotics has the potential to increase the production of organic acids by probiotic bacteria. Thus, there are several application methods to alter the GIT: the dietary introduction of acidic compounds either directly via feed supplements containing organic acids, or indirectly as a shift in fermentation originating from the presence of probiotics, prebiotics, or combined as synbiotics in the GIT. The current review aims to elaborate on the use of organic acids and organic acid stimulating dietary supplements, probiotics and prebiotics, and their subsequent effects on pathogen prevalence and the developing avian GIT microbiome.

### Antibiotics in the livestock industry

After the rapid expansion of the poultry industry in the 1940s, there was a need for basic feed components. Due to this accelerated growth in the commercial poultry industry, there was a shortage of fishmeal and other animal protein sources (8–10). With the necessity for more animal protein sources, the industry sought to determine what the Animal Protein Factor (APF), the factor in animal protein sources that promoted increased poultry performance, consisted of and to find a suitable alternative (8). APF was later discovered to be Vitamin B_12_ in 1948 (8–10). Ultimately, the search to find an effective alternative to APF helped fuel the discovery of antibiotic growth promoters (AGPs).

Alexander Fleming, an English scientist, discovered penicillin in 1928 when he was testing the ability of mold to reduce staphylococci on agar plates (8). However, it took until the early 1940s for scientists, Ernst Chain and Howard Florey, to isolate a sufficient quantity of penicillin to be tested and validated as an effective treatment for illnesses (8). Shortly after the discovery of antibiotics, a growth promoting component of fungal mycelia, an antibiotic, was observed outperforming APF, vitamin B_12_ (8, 11–14). Moore et al. included antibiotics in chicken feed and was the first research group to show an increase in weight gain due to the inclusion of antibiotics (15). Later, the use of antibiotics in feed would be coined as the term “AGPs” and be utilized for prophylactic purposes that prevented or reduced the risk for infection, as well as promoted growth in broilers.

AGPs in the poultry industry are administered in the diet when there is no clinical sign of infection, however the risk still exists. Prophylactic application of AGPs have resulted in improved weight gain, reduced bacterial presence in the GIT, reduced nutrient competition, and reduced immune stimulation (4). After the introduction of AGPs to the industry, there were concerns for the residues in meat and fungal overgrowth in animals. However, since the poultry industry does not employ antibiotics that are absorbed by the digestive tract, the concern for antibiotic residues in meat and meat products was not considered a direct concern (16, 17). As time progressed, the concerns have evolved due to consumer perception and scientific reports (8).

### Removal of antibiotics from the poultry industry

The poultry industry began to turn away from the use of antibiotics due to growing public concern over antibiotic resistant pathogens. As early as the late 1960s, the Swann Committee in the European Union (EU) researched the possibility of bacterial resistance due to the use of antibiotics in livestock diets (18). It was found in the years between 1963 and 1965 that the resistance to antibiotics could be transferable to other bacteria, as was seen in the epidemic of antibiotic resistant *Salmonella* Typhimurium (18). The epidemic of *S*. Typhimurium led the United Kingdom (UK) government to appoint the Swann Committee to monitor and identify possible resistance of pathogenic bacteria to antibiotics from animal origins (18). The Swann Committee later recommended in 1969 that the antibiotics used as growth promoters in feed diets be those that “have little or no application as therapeutic agents in man or animals and will not impair the efficacy of a prescribed therapeutic drug or drugs through the development of resistant strains of organisms” (18). The Swann Committee in that same statement deemed the use of chlortetracycline, oxytetracycline, penicillin, tylosin, and the sulphonamides as unsuitable for growth promotion (18). The statement was later adopted by the UK in 1998 (19). As the continued concerns grew in the UK and across the world, the poultry industry experienced extreme pressure to terminate the use of AGPs in the diet of poultry and other livestock.

The first country in the EU to officially ban the use of AGPs was Sweden in 1985 (18). Sweden, after joining the EU in 1995, heavily campaigned for the termination of the use of antibiotics as growth promoters in animal feed in the EU (18). In 1996, the United States (US) implemented the National Antimicrobial Resistance Monitoring System (NARMS), which monitored the antimicrobial resistance in bacteria (8). Within that same time period (1997 and 1998), the World Health Organization (WHO) and Economic and Social Community of the European Union deemed the use of antimicrobials in food animals as a public health concern, citing risks to the long-term use of antibiotics, such as resistance to antibiotics (17).

The EU finalized the ban on AGPs with the creation of Regulation 1831/2003 which eliminated the use of all AGPs as of January 1, 2006 (17). Although the overall use of antibiotics has decreased by 55% from 1986 to 1999 in conjunction with a low prevalence of antimicrobial resistance (20), there is still concern for the increase in use of therapeutic antibiotics due to the increase in infections (21).

Current concerns over antibiotic resistance have been backed by the prevalence of antibiotic resistance stemming from livestock origin. Poultry have been linked to the resistance of *Campylobacter* and *Salmonella* to multiple antibiotics. For example, a few years after the introduction of fluoroquinolones in The Netherlands, there was an increase in fluoroquinolone-resistant *Campylobacter* of poultry origin (22). The EU also experienced gentamicin resistance in *Campylobacter* from broiler meat origins that ranged from 0 to 6.3% (23). The US has seen *Campylobacter coli* resistance to gentamicin increase from 1% in 2007 to 18% in 2011 from chicken meat isolates and an increase from 1 to 6% between 2007 and 2011 from chicken isolates at slaughter (24). In addition, *Salmonella* spp. have been noted to develop a multi-drug resistance to antibiotics such as tetracyclines, sulfonamides, streptomycin, kanamycin, chloramphenicol, and some β-lactam antibiotics (25–27). However, there has been a relatively stable reporting of resistance among these antibiotics since 1996 (4). The resistance to other antibiotics has increased relatively, as seen in amoxicillin/clavulanic acid and ceftiofur, which have been associated with increases from <2 to 15% from 1998 to 2005, respectively (28).

Currently, the US poultry industry has initiated phasing out AGPs partly due to the increase in consumer concern over the usage of AGPs and the increase in AGP free exportation requirements. However, numerous growers have observed an increase in “dysbacteriosis,” a condition in which the small intestines' experience bacterial overgrowth (3). The solution is to find alternatives with similar effects as AGPs such as: (1) reducing the number of incidences and the amplitude of subclinical infections; (2) reducing the use of nutrients by bacteria; (3) improving absorption through the thinning of the intestinal wall; and (4) by reducing the amount of “growth-depressing metabolites” produced by Gram-positive bacteria (3).

## Alternatives to antibiotics: organic acids

Several alternatives have been proposed to replace AGPs in the poultry industry including exogenous enzymes, competitive exclusion products, prebiotics, probiotics, herbs, essential oils, acidic compounds, and bacteriophages (3, 29). Currently, the more common alternatives applied in broiler diets are prebiotics, probiotics, and organic acids. All are utilized with the ultimate goal of ameliorating the condition of the poultry GIT by mitigating the presence of enteric bacteria present in the GIT and improving the performance of the bird (29). It is of interest to determine how each alternative product specifically achieves improvement in bird gut health. Both organic acids and probiotics appear to have similar mechanistic impacts on bird health as many probiotics improve the physiology and anatomical structure of the intestinal cell wall, enhancement of immunological functions in the GIT, and the increased resistance to enteropathogenic bacteria activity (3). This occurs either by direct introduction of organic acids including short chain fatty acids (SCFA) in the feed or in the case of probiotic bacteria generating SCFA, hydrogen peroxide, and intermediary metabolites with antimicrobial activity once they become established in the GIT (3). Organic acids include not only SCFAs but also lactic and formic acids as well as longer carbon chain acids. Prebiotics are also of interest, as they stimulate the proliferation and maintenance of beneficial bacteria such as *Lactobacillus*, which in return increases the production of SCFA (30). Thus, organic acid, probiotic, and prebiotic supplements are interlinked because of their role in the production of SCFA and other fatty acids (31–33).

Organic acids are organic compounds that retain acidic properties (5). Most organic acids consist of carboxylic acids (-COOH). Organic acids are primarily composed of SCFAs (≤C6), also commonly referred to as volatile short-chain fatty acids (VSCFA), such as fumaric, propionic, acetic, lactic, butyric, and others. Other organic acids consist of medium-chain fatty acids (MCFA; C7 to C10), and long-chain fatty acids (LCFA; ≥C11) (Table 1).

**Table 1:** A list^1^ and description of straight-chain monocarboxylic acids^2,3,4^ and their derivatives^5^.

**Acid**	**Chemical name**	**Formula**	**MW**	**pKa**
Formic^2^	Formic Acid	HCOOH	46.03	3.75
Acetic^2^	Acetic Acid	CH_3_COOH	60.05	4.76
Propionic^2^	2-Propanoic Acid	CH_3_CH_2_COOH	74.08	4.88
Butyric^2^	Butanoic Acid	CH_3_CH_2_CH_2_COOH	88.11	4.82
Lactic	2-Hydroxypropanoic Acid	CH_3_CH(OH)COOH	90.08	3.83
Sorbic	2,4-Hexandienoic Acid	CH_3_CH:CHCH:CHCOOH	112.14	4.76
Fumaric	2-Butenedioic Acid	COOHCH:CHCOOH	116.07	3.02
Caproic^2^	1-Hexanoic Acid	CH_3_CH_2_CH_2_CH_2_CH_2_COOH	116.16	4.88
Malic	2-Hydroxybutanedioic Acid	COOHCH_2_CH(OH)COOH	134.09	3.40
Caprylic^3^	1-Octanoic Acid	CH_3_CH_2_CH_2_CH_2_CH_2_CH_2_CH_2_COOH	144.21	4.89
Tartaric	2,3-Dihydroxy-Butanedioic Acid	COOHCH(OH)CH(OH)COOH	150.09	2.93
Capric^3^	Decanoic Acid	CH_3_(CH_2_)_8_COOH	177.26	4.90
Citric	2-Hydroxy-1,2,3-Propanetricarboxylic Acid	COOHCH_2_C(OH)(COOH)CH_2_COOH	192.14	3.13
Lauric^4^	Dodecanoic Acid	CH_3_(CH_2_)_10_COOH	200.32	5.30

1 *Adapted from Dibner and Buttin and Cherrington et al. (6, 34)*.

2 *Classified as a short-chain fatty acid (SCFA; ≤C6)*.

3 *Classified as a medium-chain fatty acid (MCFA; C7-C10)*.

4 *Classified as a long-chain fatty acid (LCFA; ≥C11)*.

5 *Derivatives of saturated straight chain fatty acids: unsaturated (sorbic), hydroxylic (citric, lactic), multicarboxylic (fumaric, malic, tartaric, and citric)*.

Due to the lipophilic nature of LCFA, their antimicrobial properties may be a constituent of their potential to incorporate themselves into target cell membranes and promote leakage of cellular protons or ions, such as in Gram-positive bacteria (35–37). However, it has been demonstrated by Shue and Freese that the resistance possessed by Gram-negative species to MCFA and LCFA is in part due to the presence of the lipopolysaccharide (LPS) layer in the cell wall (38). Thus, LPS prevents MCFA and LCFA from crossing the cell membrane and into the cell (34). Further, Gram-negative bacteria, such as *E. coli*, possess the ability to assimilate MCFA and LCFA into the cell and subsequently metabolize them per the β-oxidation cycle (39).

Alternatively, specific bacterial groups such as *Salmonella* and *Escherichia coli* are capable of utilizing SCFA as energy sources (39–43), whereas fermentative bacteria produce organic acids when oxygen is not available (29). More specifically, acetic acid is a source of carbon and energy for bacteria by activating enzymes of the glyoxylate pathway, isocitrate lyase, and malate synthase (39). Furthermore, lactobacilli, streptococci, lactococci, and enterococci are all capable of fermenting sugars to produce lactate; however, if sugar is scarce these bacteria are capable of generating acetate, formate, and ethanol from fermentation to enhance ATP production (44).

Organic acids were initially added to feed for sanitization purposes such as to reduce fungal contamination in feed and as a preventative against salmonellosis in poultry (45–47). However, in the past 30 years, formic and propionic acid have been examined for bactericidal activity, *in vivo*, of poultry (48). Organic acids utilized in feed are not only capable of decontaminating feed but have the potential to reduce enteric bacteria internally in poultry.

Weak organic acids (C1-C7) with a pKa between 3 and 5 are explicitly used for their antimicrobial activity (5). There are two major types of organic acids (Table 2). The first group (lactic, fumaric, citric) are capable of generally lowering the pH of the stomach, thus reducing the acid sensitive bacteria present indirectly. The second group (butyric, formic, acetic, propionic, and sorbic) lower the pH in the GIT by directly acting upon the cell wall of Gram-negative bacteria (5, 49). Organic acids ameliorate the conditions of the GIT through the reduction of GIT pH, promoting proteolytic enzyme activity and nutrient digestibility, intensifying pancreatic secretions, encouraging digestive enzyme activity, creating stability of the microbial population and stimulating the growth of beneficial bacteria, and by being bacteriostatic and bactericidal to pathogenic bacteria (5). With the need to find a suitable alternative to AGPs, a wide range organic acids have been utilized in poultry diets for the potential to mitigate pathogen prevalence in the GIT of poultry.

**Table 2:** Two different mechanisms of organic acids on altering the pH of the gastrointestinal tract (GIT) and its subsequent effect on pathogens.

**Acids**	**Effect**
Lactic, fumaric, citric	Indirectly mitigating or eliminating pathogens by decreasing the environmental pH in the GIT^1^.
Butyric, formic, acetic, propionic, and sorbic	Directly mitigating or eliminating pathogens by acting upon the cell wall of Gram-negative bacteria and subsequently lowering the pH in the GIT^1^.

1 *Adapted from Papatisiros et al. and Diener et al. (5, 49)*.

## Organic acids as feed additives

As previously mentioned, organic acids can benefit poultry internally by their ability to lower the pH of the gastrointestinal tract. It has been found that organic acids such as fumaric, propionic, lactic, and sorbic acid have the ability to reduce the colonization of pathogenic bacteria and the production of toxic metabolites through acidification of the diet (50). Although the crop and gizzard are the locations in which propionic and formic acid are confined to, the crop is one of the initial locations for *Salmonella* establishment that can lead to subsequent infection of the bird (51). It has also been demonstrated that most *Salmonella* spp. are killed when the pH value is the equivalent to that of the crop and proventriculus, *in vitro* (52). In addition, the vertical transmission and initial colonization of chicks with *Salmonella* can be reduced through the dietary inclusion of organic acids (53). Although organic acids can indirectly have an impact on pathogenic bacteria by lowering the pH of the GIT, they can also elicit non-pH direct toxic effects on bacterial metabolism.

Although the most noted benefit of organic acids is its ability to lower the pH of the GIT, organic acids can also prevent pathogen livability on the cellular level. Organic acids possess the ability to target the cell wall, cytoplasmic membrane, and particular functions of metabolism in the cytoplasm associated with replication, protein synthesis, and function (48, 54). VSCFA, consisting of weak organic acids that are bacteriostatic without affecting intestinal microbiota, are not regarded as acidifiers as their mode of action is to directly diffuse across the cell membrane of bacteria in the undissociated form without lowering the bowel pH (55). VSCFA, once diffused across the bacterial cytoplasm, lower the internal pH of the bacteria (55).

The specific effectiveness of a particular organic acid relies heavily on several factors such as: type and acidity of the SCFA, inclusion rate of acids, diet composition and buffering within the diet, level of “intraluminal production of acids” by lactic acid producing bacteria (LAB) in GIT, feed palpability, receptor on the epithelial villi for bacterial colonization, vaccinate immunity, welfare, and age (5).

Some concerns for the use of organic acids include their inability to affect the lower part of the GIT, bacteria's ability to create a resistance against organic acids, and their hindering effect on host beneficial bacteria such as LAB. Much of their bacterial impact is related to their effective concentration present in different compartments of the GIT. For example, Thompson and Hinton noted that as SCFAs move along the digestive tract, their concentration decreases due to digestion and metabolism (51). It has also been reported by Hume et al. that most of the propionic acid that was in the treated feed did not get past the crop, proventriculus, and gizzard and thus never reached the small intestines (48, 56). Most organic acids will dissociate before reaching the lower GIT and thus having little to no effect on the GIT (56). Although it was stated earlier the initial site of *Salmonella* colonization is the crop, it is important for organic acids to enter the lower GIT, as the ileum and ceca are also considered primary sites of infection. Furthermore, it is unlikely for organic acids to prevent a large infectious dose of *Salmonella* from getting past the crop (51).

To combat the potential decrease in effective concentrations of organic acids as they traverse the GIT, encapsulation of organic acids offers the potential to not only protect them but control their subsequent release as they pass through the poultry GIT. It has been demonstrated that the dietary inclusion of encapsulated butyric acid has the capability to improve digestion and absorption (57), reduce the infection of *S*. Enteritidis throughout the GIT (55) and reduce stress-induced catabolism and oxidative injury of tissues (58) of broilers.

## Organic acids produced by introduction of probiotics and prebiotics to the GIT

Although SCFA can be experimentally provided as feed additives in poultry diets, they are also naturally produced by the GIT, and their relative concentrations and types can be altered. In chickens, ruminants, and humans the production of SCFA in the GIT has been reported as high as 190 mM (59–62). Research has demonstrated that both probiotic and prebiotics stimulate the production of SCFAs in the GIT of poultry (3, 63–66) either through the direct production of SCFA by lactic acid producing bacteria (LAB), a type of probiotic, or through the administration of prebiotic substances which increase the presence of LAB and their production of SCFA (67, 68). Thus, probiotic and prebiotic supplementation can enhance SCFA production and, in turn, their impact on the avian microbiome. The following subsection provides discussion of specific studies that illustrate this impact.

### Probiotics and their influence on SCFA production

Lilley and Stillwell originally conceived the term probiotics as “a substance produced by one microorganism which stimulated the growth of another” in 1965, well after the discovery of antibiotics (69). Although the term was not coined until after the discovery of antibiotics, probiotics had been around since the early Twentieth century (70). Over time as more knowledge was obtained on the nature of what represented a true probiotic culture, the definition started to change to define their usefulness and application better. In 1989, the definition was modified to a “live microbial feed supplement which beneficially affects the host animal by improving its intestinal microbial balance” by Fuller (70). Three years later in 1992, Havenaar and Huis in't Veld extended the definition to “a mono or mixed culture of live microorganisms which, applied to animal or man, affect the host beneficially by improving the properties of the indigenous microflora” (70, 71). The definition of probiotics has now been established by Fuller as “a preparation consisting of live microorganisms or microbial stimulants which affects the indigenous microflora of the recipient animal, plant or food in a beneficial way” (70).

Microorganisms that have been considered as probiotics include: lactic acid producing bacteria, avirulent mutants of *E. coli, Clostridium difficile, S*. Typhimurium, yeasts, fungi, viruses, and bacteriophages (70). Current research using probiotic bacteria include: *Bifidobacterium, Lactobacillus, Bacillus, Enterococcus, Streptococcus, Pediococcus*, and *Saccharomyces* (5). Probiotics serve to protect the GIT microbiota through bacterial antagonism, bacterial interference, barrier effect, competitive exclusion, and colonization resistance (70).

The most common probiotic spp. utilized in poultry diets are from the genera *Lactobacillus, Enterococcus, Pediococcus*, and *Bacillus;* however, extensive research has been conducted on *Lactobacillus* species (63, 72, 73). Further, various probiotic spp, differ in their ability to colonize the GIT of animals. Those that are considered colonizing species are *Lactobacillus* and *Enterococcus* spp., while *Bacillus* spp. and *Saccharomyces cerevisiae* are free-flowing and do not colonize the GIT (3). As stated earlier, the benefits of probiotic supplementation include: onset of changes of the physiology and anatomical structure of the intestinal cell wall; enhancement of immunological functions in the GIT; and the enhanced resistance to enteropathogenic bacteria (3). These actions are typically accomplished via coupling with the production of SCFA, hydrogen peroxide, and intermediary metabolites with antimicrobial activity (3).

Probably the best-characterized group of probiotics are lactic acid producing bacteria (LAB), such as *Lactobacillus*. LAB generate lactic acid *in vitro* and the lactic acid produced is utilized for the production of butyric acid by Clostridial clusters, which supports the concept of cross-feeding (3). *Lactobacillus* spp. have been found to reduce pathogenic attachment to the ileal epithelial cells through exclusion and competition (72). *Lactobacillus* also elicits antibacterial effects by producing lactic acid (63). Lactic acid, an organic acid, can lower the GIT pH, thus creating a hostile environment for resident pathogenic bacteria. *Lactobacillus acidophilus* is found to be the most sufficient candidate as a dietary appurtenance (71). *L. acidophilus* has the potential to decrease the external pH to lower values than other lactic acid producing bacteria and can reach a medium pH of 3.5 (64). Thus, *Lactobacillus* spp. are considered excellent candidates as AGP alternatives.

### Prebiotics influence on SCFA production

Prebiotics have also been considered as valid AGP alternatives (74). Prebiotics are described as beneficial non-digestible feed ingredients that when fed selectively enhance populations of bacteria in the GIT (59). More recently, prebiotics have been described as “a substrate that is selectively utilized by host microorganisms conferring a health benefit” (75). Thus, prebiotics influence the GIT by acting as substrates for beneficial bacteria. Prebiotics include non-digestible carbohydrates such as oligosaccharides and polysaccharides, particular peptides, proteins, and specific lipids (76). Poultry research investigating the application of prebiotics as antibiotic alternatives typically revolves around the administration of oligosaccharides which include mannanoligosaccharides (MOS) galactooligosaccharides (GOS), and fructooligosaccharides (FOS) (32, 33, 75, 77–79).

The dietary inclusion of prebiotics has been demonstrated to influence the microbial profiles of the avian GIT. Kim et al., reported the increased concentration of lactobacilli at the the ileal cecal junction of 4 week old broiler chickens fed diets containing 0.25% FOS and 0.25% MOS and a decrease in the populations of *Clostridium perfringens* in birds fed diets containing 0.25% FOS, 0.05% MOS, and avilamycin (80). The dietary inclusion of MOS (5 g/kg) and FOS (5 g/kg) have also been shown to change the jejunal, ileal, and cecal *Lactobacillus* community profiles of 25 d Cobb 500 broilers, with differences in *Lactobacillus* communities being noted between MOS and FOS treated broilers (81). Although research has demonstrated the effect prebiotics have on bacteria, performance has been shown to not be improved by the dietary inclusion of MOS or FOS (80, 81). In contrast, the addition of Bio-Plus 2B® into diets of Ross 308 broilers improved the feed conversion ratio (FCR) throughout the entirety of the study (42 d) compared to those fed control diets (82).

In addition, the dietary supplementation of prebiotics has demonstrated the enhanced production of organic acids in the GIT. The dietary inclusion of FOS has demonstrated the ability to increase populations of *Bifidobacterium* and *Lactobacillus* in the intestinal and cecal digesta of 49 d old male Avian Farms broilers (83). Thus, the increase of LAB species such as *Lactobacillus* and *Bifidobacterium* may enhance the production of SCFA in the GIT. Furthermore, *Lactobacillus rhamnosus, Lactobacillus acidophilus, Bifidobacterium longum*, and *Bifidobacterium bifidus* in the presence of millet dietary fibers have exhibited the ability to produce SCFAs such as acetate, propionate, and butyrate, *in vitro*, with the production of acetate being most significant (84). A study investigating the inclusion of inulin (1%)in the diets of 42 d old Cobb 500 broilers reported the increased concentration of acetate in the jejunum and an increase in the proportions of n-butyrate and n-valerate in the cecal digesta in broilers fed diets containing inulin compared to those fed control diets (85). In short, the dietary supplementation of prebiotics appears to contribute to the increased production of SCFA *in situ*.

## Mechanisms of organic acids vs. lactic acid producing bacteria

Research has demonstrated that both organic acids and LAB have the capability to improve broiler performance and reduce pathogenic bacteria (86–90). Since the modes of action for both organic acid supplements and LAB involve the lowering of the pH of the GIT, many of their benefits appear to be similar. However, LAB and organic acids should also still be considered in some respects considerably different in their effectiveness, mechanisms, and interaction with one another.

Although LAB do not directly destroy enteric bacteria, LAB are able to inhibit colonization and further growth and establishment of pathogenic bacteria. Furthermore, LAB byproducts beyond SCFAs, such as hydrogen peroxides, and intermediary metabolites also contribute to the reduction of pathogens present in the GIT. In fact, research has demonstrated when *S*. Enteritidis at 10^6^ CFU and *L. salvarius* at 10^8^ CFU were gavaged orally and simultaneously into the proventriculus of 1-day old broiler chicks, at 21 days of age all birds were negative for *Salmonella* (91). It has been noted that SCFAs when interacting with Gram-negative bacteria are not only bacteriostatic but can also be bactericidal (51). Furthermore, organic acids such as SCFAs are produced in millimolar concentrations in the GIT of animals due to the prevalence of anaerobic bacteria. Organic acids, being SCFA, also possess the ability to lower the pH of the GIT and improve broiler performance similar to LAB. Thus, previous research has seen both methods to be beneficial in the reduction of pathogenic bacteria (86–90).

Another challenge of using organic acids as an alternative to AGP is the resistance bacteria can develop to stressful environments. It has been reported that *E. coli* and *Salmonella* can elicit a tolerance to environments that induce stress such as an acidic environment created by the use of organic acids (48). In addition, Diez-Gonzalez and Russell have reported the increased resistance to extreme acidic conditions of *E. coli* O157:H7 after exposure to SCFAs (92). Likewise, Conner and Kotrola previously observed that *E. coli* exhibited the ability to live in acidic condition (pH ≥ 4.0) below 4.0°C and for up to 56 d, however, the temperature and type of acidifier affect their survival (93). In addition, pH-independent tolerance is also possible. For example, Kwon and Ricke reported the increased acid resistance displayed by *S*. Typhimurium occurred after exposure to a single SCFA at high concentration but neutral pH (94). Furthermore, it has been reported that the proportions of SCFAs within the large intestines can influence the cross-resistance of *S*. Typhimurium 14028s to other stressors such as an extreme pH (pH 3.0), 2.5 M NaCl, and 20 mM H_2_O_2_ (95).

Not only can bacteria build a resistance to organic acids, but pathogenic bacteria can also lower their internal pH to protect themselves from the acidic properties of organic acids, thus rendering them ineffective in being bactericidal against pathogenic bacteria (48). Furthermore, fermentative bacteria have the ability to lower their intracellular pH in the event that the extracellular pH becomes highly acidic. If the intracellular pH is lowered, the bacterium has a much smaller pH gradient across the cell membrane and will be protected from anion accumulation (53).

The most significant challenge to organic acid feed additive use is their potentially detrimental effect on LAB. In previous research, the use of organic acids in the diet reduced not only the amount of lactic acid but the LAB present in the GIT. As early as 1989, Impey and Mead reported that adding 1.0% formic acid into a food slurry containing *Salmonella* and *Lactobacilli*, not only killed *Salmonella* but *Lactobacilli* as well (pH < 4.0; 37°C) (96). It was also observed by Hume et al. that organic acids reduced LAB (56). The finding by Hume et al. is consistent with the conclusion by Thompson and Hinton that LAB were reduced by the inclusion of organic acids (51, 56). In one of the studies conducted by Thompson and Hinton, 68% formic acid and 20% propionic acid product, was added to a poultry diet and resulted in an increase of propionic and formic acid, as well a decrease in lactic acid in the crop (51). This interaction suggests that propionic and formic acid inhibit LAB and thus reduced lactic acid. Consequently, SCFA may be counterproductive to the overall development of microbiota in the GIT of broiler chickens.

Poultry are born without an established microbiota in their GIT (97) and are removed from maternal care to be incubated in a controlled environment. Thus, poultry housed in modern production facilities have difficulty in establishing beneficial microbiota associated with a mature GIT microbiota (70). This corresponds with research where intestinal infections affect germ-free animals more than those with an established microbiota (98, 99). Probiotics have been especially important in improving the microbiota composition of poultry, as well as protecting poultry from intestinal infections and are recognized as an alternative to AGPs. If the use of organic acids in poultry reduces the concentration of LAB present in the GIT, it could increase the chance of *Salmonella* colonizing the GIT. This especially could occur as organic acids are limited to the crop and may not be able to handle a high inclusion of *Salmonella* (51). Probiotics (LAB) serve to protect the GIT microbiota through bacterial antagonism, bacterial interference, barrier effect, competitive exclusion, and colonization resistance (70). LAB are not only beneficial in protecting the bird from pathogens but also provides the bird with physical enhancements to the GIT. These enhancements include strengthening the gut wall integrity, enhance anti-inflammatory response, and correct dysbacteriosis (29). With all of the benefits that LAB provide to poultry, it is vital to ensure their survival and utilization in poultry.

As both organic acids and LAB are potential alternatives to AGPs, it is imperative to understand the specific effect attributable to each method that can be associated with bird performance and welfare, as well as the interactions they have on one another within the bird GIT.

## SCFA and potential application of avian microbiome research technologies

Due to current poultry industry practices, prior to hatch, the GIT of chicks are presumed relatively sterile (100). However, immediately after hatch, the chick's microbiome begins to develop as the colonization of the GIT occurs until a diverse and dynamic microbiome is established (101). Previously, research has indicated that on day 0 (post-hatch) of age the cecal microbiome of broilers consists of 50 genera, whereas, by 42 days of age the cecal diversity is increased to over 200 genera (102). Additionally, shortly after post-hatch, the chick's nutrient source is shifted from the yolk to the carbohydrate- and protein-based diet (103, 104). The shift in the nutrient source is accompanied by the rapid development on the GIT and associated organs which can be directly affected by the gut microbiome (105, 106) Thus, it is imperative to alter the GIT microbiome at an earlier age, before what would be considered the adult diverse microbiome becomes stabilized.

Currently, the application of next-generation sequencing technologies to delineate the gut microbiome of poultry is becoming more routine and this, in turn, has resulted in an enhanced understanding of how bacteria of the GIT may influence the development and performance of poultry. The prominent phylum in the crop, gizzard, small intestines, and ceca is the bacterial phylum Firmicutes [Table 3; (107, 112, 113)]. The proportion of Firmicutes, primarily Lactobacilli, has been reported to be >90% in the GIT (112, 114). Thus, the microbiome of the small intestines consists mainly of *Lactobacillus, Enterococcus*, and *Clostridiaceae* species (97, 112, 115–118). However, the greatest diversity and quantity of bacteria is located within the ceca, where microbial fermentation is also the most active (112). The ceca are characterized by possessing a high proportion of Firmicutes, 50–90% of all taxa (107, 113). The predominant phyla in the ceca have been reported as Bacteroidetes (23–46 %), Proteobacteria (1–16 %), and Archaea (0.81 %) (107–110).

**Table 3:** Predominant phyla and bacterial population in the chicken gastrointestinal tract^1^.

**Gastrointestinal segment**	**Phylum**	**Genus**
Crop	Firmicutes	*Lactobacillus*
	Actinobacteria	*Bifidobacterium*
	Proteobacteria	*Enterobacter*
Proventriculus	Firmicutes	*Acetanaerobacterium, Clostridium, Faecalibacterium, Lactobacillus, Megamonas, Peptococcus, Pseudobutyrivibrio, Ruminococcus, Sporobacter, Subdoligranulum*
	Fungi	*Candida*
Ventriculus	Firmicutes	*Lactobacillus, Enterococcus*
Small Intestine	Firmicutes	*Candidatus Arthromitus, Clostridium, Lactobacillus, Ruminococcus*
	Proteobacteria	*Enterococcus, Escherichia*
Ceca^2^	Bacteroidetes	*Bacteroides*
	Proteobacteria	*Bilophila, Escherichia*
	Archea	*Methanobrevibacter, Methanobacterium, Methanococcus, Methanopyrus, Methanosphaera, Methanothermobacter, Methanothermus*,
Large Intestines	Fimicutes	*Lactobacillus*
	Proteobacteria	*Escherichia, others*

1 *Data was adapted from Qu et al. (107), Saengkerdsub et al. (108, 109), Gong et al. (110), and Yeoman et al. (111)*.

2 *Taxa of the ceca was constricted to the most pertinent phyla and genera although many more have been described*.

The GIT microbiome has a fundamental role in the production of SCFA (119). The ceca, especially, generate SCFA through various fermentation pathways and may recover up to 10% of energy available in the diet (120, 121). In the ceca, a vast majority of the Families within the phylum Firmicutes belongs are members of the Clostridiales, a significant component of SCFA metabolism (107, 122). In the ceca, SCFA production is derived from the hydrolysis and fermentation of non-starch polysaccharides [NSP; (123)].

The bacterial fermentation of NSPs in the ceca has been reported to consist primarily of acetic acid, propionic acid, and butyric acid (124). Sergeant et al. identified fermentation pathways encoded in the cecal metagenome that are responsible for the production of acetate, propionate, and butyrate (125). The authors identified gene clusters, encoding enzymes methylmalonyl-CoA epimerase and methylmalonyl-CoA decarboxylase, from Bacteroidetes and Firmicutes to be involved in the production of propionate in the chicken ceca (125). Further, Sergeant et al. speculated the involvement of *Megamonas* and *Dialister* in a novel propionate fermentation pathway (125). The butyrate fermentation pathway is encoded in the BCD/ETF complex and phosphotransbutylase/butyrate kinase genes of butyrate-producing bacteria and sequences are reported to be from Bacteroidetes (125).

There is limited research investigating the effect organic acids have on the GIT microbiome of poultry. Early research demonstrated the negative correlation between Enterobacteriaceae and organic acids such as acetate, butyrate, and propionate in the ceca of broilers (126). More recently, Oakley et al. supplied organic acids as a dietary feed additive (propionic acid and MCFA), in the water supply (formic acid, propionic acid, ammonium formate, MCFAs, an emulsifier, and, propylene glycol), or a combination of the two and examined the subsequent change in the cecal microbiome of Ross × Cobb male broilers over a 42-d period (102). Oakley et al. reported that treatment had little to no effect on the cecal microbiome (102). Instead, the authors demonstrated that the drastic changes in the cecal microbiome occurred as a function of bird age (102) which agrees with the increase in cecal microbiome diversity with age observed by others (127–130). Furthermore, Oakley et al. identified the cecal microbiota to primarily consist of *Flavonifractor, Pseudoflavonifractor*, and *Lachnospiracea* on d 7, *Faecalibacterium* (23–55 % of sequences) on d 21, and *Faecalibacterium* and *Roseburia* on d 42 (102). Also, on d 42, *Lachnospiracea incertae sedis* and *Oscillibacter* were recorded as being abundant (102). Some members of *Lachnospiracea incertae sedis* and *Oscillibacter* have been identified as SCFA producers (131, 132). In another study, the dietary supplementation of a microencapsulated feed additive consisting of a phenolic essential oil, thymol, and an organic acid, sorbic acid, resulted in the decrease of *Campylobacter jejuni* and a reduction in the abundance of *Streptococcus* in Ross 308 broilers inoculated with 10^4^ CFU of *C. jejuni* (A2008a and G2008b) (133).

Although the microbial diversity in the ceca increases with age (102, 127–130), it has been demonstrated that organic acids reach their highest concentrations in the GIT of broilers on d 15 (126). As mentioned previously, SCFA production can be enhanced with the dietary supplementation of prebiotics (83–85). The increase in SCFA production is a consequence of the increased colonization of LAB species within the GIT. Birds fed diets containing FOS have been reported to alter the microbiome by enhancing the production of *Bifidobacterium* and *Lactobacillus* which in return enhance digestive enzyme activity and suppress pathogens such as *E. coli* (83). Therefore, by increasing the population of LAB or by supplementing diets with SCFA, the concentration of SCFA in the GIT may be enhanced beyond the peak experienced on day 15.

Future studies should be aimed at evaluating the potential of novel feed additives, organic acids, probiotics, prebiotics, on their potential to change the concentration of specific microbiome populations that may enhance or hinder the performance of poultry. More specifically, studies should focus on the change of *Enterobacteriaceae*. A negative correlation between the concentration of SCFAs such as acetate, propionate, and butyrate and the concentration of *Enterobacteriaceae* has been observed in the ceca of broilers (133). Furthermore, the ratio of Firmicutes to Bacteroides has been identified as a potentially significant index due to its possible correlation with performance. In mice, it has been reported that an increase in Bacteroidetes has been linked to a decrease in nutrient absorption, while the increase in Firmicutes has resulted in an increase in nutrient absorption (134). Another population for monitoring is *Lactobacillus* spp. as their presence in the lower small intestines has been associated with reduced performance (135).

## Conclusions

As the poultry industry is faced with increased demand for ABF, an alternative to antibiotics needs to be identified that enhances the GIT microbiome of poultry. It is also crucial that this alternative is easily integrated into nutrition, genetics, housing, and veterinarian care for future application. Thus, it is imperative for research to be conducted to determine the most effective method in reducing pathogenic bacteria in the gut, improving broiler performance, and improving gut morphology. To accomplish this will require the application of methodologies that increase the understanding of the avian GIT microbiota. Indeed, the availability of microbiome sequencing offers opportunities to characterize the poultry GIT microbial community in response to organic acids. However, it will be essential to profile GIT populations along the entire GIT from crop to ceca to get a better understanding of where organic acids are eliciting their effects and how this influences bird performance and control of pathogen colonization in the GIT. Once more becomes understood, it should be possible to develop more precisely targeted strategies for employing organic acids as feed additives and eventually optimizing multiple hurdle combinations of probiotics and organic acid combinations.

## Author contributions

DD contributed thoughts and wrote the review. AK contributed financial support of the graduate stipend of the first author, DD, and supervised the development and revision of this review with the assistance of SR.

### Conflict of interest statement

The authors declare that the research was conducted in the absence of any commercial or financial relationships that could be construed as a potential conflict of interest.

## References

[1] Cervantes HM Antibiotic-free poultry production: is it sustainable? J Appl Poult Res. (2015) 24:91–7. 10.3382/japr/pfv006

[2] Cheng G Hao H Xie S Wang X Dai M Huang L. Antibiotic alternatives: the substitution of antibiotics in animal husbandry? Front Microbiol. (2014) 5:217. 24860564 10.3389/fmicb.2014.00217 PMC4026712

[3] Huyghebaert G Ducatelle R Van Immerseel F An update on alternative to antimicrobial growth promoter for broilers. Vet J. (2010) 187:182–8. 10.1016/j.tvjl.2010.03.003 20382054

[4] Economou V Gousia P. Agriculture and food animals as a source of antimicrobial-resistant bacteria. Infect Drug Resist. (2015) 8:49–61. 10.2147/IDR.S55778 25878509 PMC4388096

[5] Papatisiros VG Katsoulos PD Koutoulis KC Karatzia M Dedousi A Christodoulopoulos G Alternatives to antibiotics for farm animals. CAB Rev Ag Vet Sci Nutr Res. (2013) 8:1–15. 10.1079/PAVSNNR20138032

[6] Dibner JJ Buttin P Use of organic acids as a model to study the impact of gut microflora on nutrition and metabolism. J Appl Poult Res. (2002) 11:453–63. 10.1093/japr/11.4.453

[7] Dibner J Alimet feed supplement: value beyond methionine. Feedstuffs (2003) 44:12–6.

[8] Jones FT Ricke SC. Observations on the history of the development of antimicrobials and their use in poultry feeds. Poult Sci. (2003) 82:613–7. 10.1093/ps/82.4.613 12710481

[9] Ott WH Rickes EL Wood FR. Activity of crystalline vitamin B12 for chick growth. J Biol Chem. (1948) 174:1047–48. 18871266

[10] Rickes EL Brink NG Koniuszy FR Wood TR Folbers K. Crystalline vitamin B12. Science (1948) 107:396–7. 10.1126/science.107.2781.396 17783930

[11] Stokstad ELR Jukes TH Pierce J Page AC Franklin AL. The multiple nature of animal protein factor. J Biol Chem. (1949) 180:647–54. 18135798

[12] Hill DC Brannion HD The use of an animal protein factor supplement in practical poultry ration. Poult Sci. (1950) 29:405–8. 10.3382/ps.0290405

[13] Sunde ML Cravens WW Elvehjam CA Halpin JG An unidentified factor required by chicks fed practical rations. Poult Sci. (1950) 29:204–7. 10.3382/ps.0290204

[14] Swenson HJ Effect of a vitamin B12 concentrate and liver meal on growth and feed efficiency of chicks fed an all plant protein ration. Poult Sci. (1951) 30:55–62. 10.3382/ps.0300055

[15] Moore PR Evenson A Luckey TD McCoy E Elvehjem EA Hart EB Use of sulphasuccidine, streptothricin and streptomycin in nutrition studies with the chick. J Biol Chem. (1946) 165:437–41. 20276107

[16] Donoghue DJ. Antibiotic residues in poultry tissues and eggs: human health concerns? Poult Sci. (2003) 82:618–21. 10.1093/ps/82.4.618 12710482

[17] Castanon J. History of the use of antibiotic as growth promoters in european poultry feeds. Poult Sci. (2007) 86:2466–71. 10.3382/ps.2007-00249 17954599

[18] Doeschate RAHM Raine H History and current use of feed additives in the European Union: legislative and practical aspects. In: Avian Gut Function in Health and Disease. Poultry Science Symposium Series. Vol. 28 (2006). p. 3–12.

[19] House of Lords Select Committee on Science and Technology – Seventh Report. HMSO, London (1998).

[20] Wierup M. The experience of the 1986 year ban of antimicrobial growth promoters with special reference to animal health, disease prevention, productivity, and usage of antimicrobials. Microb Drug Resist. (2001) 7:183–90. 10.1089/10766290152045066 11442345

[21] Casewell M Friis C Marco E McMullin P Phillips I. The European ban on growth-promoting antibiotics and emerging consequences for human and animal health. J Antimicrob Chemother. (2003) 52:159–61. 10.1093/jac/dkg313 12837737

[22] Endtz HP JRuijs G van Klingeren B Jansen WH van der Reyden T Mouton RP. Quinolone resistance in Campylobacter isolated from man and poultry following the introduction of fluoroquinolones in veterinary medicine. J Antimicrob Chemother. (1991) 27:199–208. 10.1093/jac/27.2.199 2055811

[23] EFSA (European Food Safety Authority) and ECDC (European Centre for Disease Prevention and Control) The European Union Summary Report on antimicrobial resistance in zoonotic and indicator bacteria from humans, animals and food in 2012. EFSA J. (2014) 12:3590 10.2903/j.efsa.2014.3590 PMC7009656 32625816

[24] Centers for Disease Control and Prevention, US Department of Agriculture, US Food and Drug Administration National Antimicrobial Resistance Monitoring System 2011 Executive Report (2011). Available online at: http://www.fda.gov/downloads/AnimalVeterinary/SafetyHealth/AntimicrobialResistance/NationalAntimicrobialResistanceMonitoringSystem/UCM407962.pdf (Cited April 10, 2018).

[25] Olsen SJ Ying M Davis MF Deasy M Holland B Iampietro L. Multidrug-resistant salmonella typhimurium infection from milk contaminated after pasteurization. Emerg Infect Dis. (2004) 10:932–5. 10.3201/eid1005.030484 15200835 PMC3323239

[26] Gebreyes WA Altier C. Molecular characterization of multidrug-resistant *Salmonella enterica* subsp. enterica serovar Typhimurium isolates from swine. J Clin Microbiol. (2002) 40:2813–22. 10.1128/JCM.40.8.2813-2822.2002 12149335 PMC120621

[27] Alcaine SD Warnick LD Wiedmann M. Antimicrobial resistance in nontyphoidal Salmonella. J Food Prot. (2007) 70:780–90. 10.4315/0362-028X-70.3.780 17388077

[28] Foley SL Lynne AM. Food animal-associated Salmonella challenges: pathogenicity and antimicrobial resistance. J Anim Sci. (2008) 86 (Suppl. 14):173–87. 10.2527/jas.2007-0447 17878285

[29] Van Immerseel F De Zytter L Houf K Pasmans F Haesebrouck F Ducatelle R Strategies to control Salmonella in the broiler production chain. W Poult Sci. (2009) 65:367–92. 10.1017/S0043933909000270

[30] Kim SH Park SY Yu DJ Lee SL Ryu KS Lee DG Effects of feeding Aspergillus oryzae ferments on performance, intestinal microbiota, blood serum, components and environmental factors in broiler. Korean J Poult Sci. (2003) 30:151–9.

[31] Herfel TM Jacobi SK Lin X Fellner V Walker DC Jouni ZE Odle J. Polydextrose enrichment of infant formula demonstrates prebiotic characteristics by altering intestinal microbiota, organic acid concentrations, and cytokine expression in suckling piglets. J Nutr. (2011) 141:2139–45. 10.3945/jn.111.143727 22013198

[32] Pourabedin M Zhao X Prebiotic and gut microbiota in chickens. FEMS Microbiol Lett. (2015) 362:1–8. 10.1093/femsle/fnv122 26208530

[33] Pourabedin M Guan L Zhao X. Xylo-oligosaccharides and virginiamycin differentially modulate gut microbial composition in chickens. Microbiome. (2015) 3:3–15. 10.1186/s40168-015-0079-4 25874109 PMC4396176

[34] Cherrington CA Hinton M Meas GC Chopra I. Organic acids: chemistry, antibacterial activity and practical applications. Adv Microb Physiol. (1991) 32:87–108. 10.1016/S0065-2911(08)60006-5 1882730

[35] Galbraith H Miller TB. Effect of long chain fatty acids on bacterial respiration and amino acid uptake. J Appl Microbiol. (1973) 36:659–75. 10.1111/j.1365-2672.1973.tb04151.x 4787613

[36] Kondo E Kanai K. Further studies on the lethal effect of long-chain fatty acids on mycobacteria. Jpn J Med Sci Biol. (1976) 29:25–37. 10.7883/yoken1952.29.25 785063

[37] Greenway DLA Dyke KGH. Mechanism of the inhibitory action of linoleic acid on the growth of Staphylococcus aureus. J Gen Microbiol. (1979) 115:233–45. 10.1099/00221287-115-1-233 93615

[38] Shue CW Freese E Lipopolysaccharide layer protection of gram-negative bacteria against inhibition by long-chain fatty acids. J Bacteriol. (1973) 115:869–75. 4199515 10.1128/jb.115.3.869-875.1973 PMC246330

[39] Nunn WD Two-carbon compounds, and fatty acids as carbon sources. In: Neidhardt DC, editor. Escherichia coli and Salmonella typhimurium. Washington, DC: American Society of Microbiology (1987). p. 285–301.

[40] Durant JA Corrier DE Byrd JA Stanker LH Ricke SC. Feed deprivation affects crop environment and modulates *Salmonella* Enteritidis colonization and invasion of leghorn hens. Appl Env Microbiol. (1999) 65:1919–23. 10223980 10.1128/aem.65.5.1919-1923.1999 PMC91277

[41] Durant JA Corrier DE Stanker LH Ricke SC. Expression of the hilA Salmonella Typhimurium gene in a poultry Salm. Enteritidis isolate in response to lactate and nutrients. J Appl Microbiol. (2000) 89:63–9. 10.1046/j.1365-2672.2000.01089.x 10945780

[42] Clark DP Cronan JE. Two-carbon compounds and fatty acids as carbon sources. In: Neidhardt FC editors. Escherichia coli and Salmonella: Cellular and Molecular. Biology Web ed. (2005). Available online at: http://www.ecosal.org/ecosal/index.jsp (Accessed April 17, 2018)

[43] Wegener WS Reeves HC Rabin R Ajl SJ. Alternate pathways of metabolism of short-chain fatty acids. Bacteriol Rev. (1968) 32:1–26. 4869938 10.1128/br.32.1.1-26.1968 PMC378289

[44] Van Immerseel F Russel JB Flythe MD Gantois I Timbermont L Pasmans F Haesebrouck F Ducatelle R. The use of organic acids to combat Salmonella in poulty: a mechanistic explanation of the efficacy. Avian Pathol. (2006) 35:182–8. 10.1080/03079450600711045 16753609

[45] Khan M Katamay M Antagonistic effects of fatty acids against Salmonella in meat and bone meal. Appl Microbiol. (1969) 17:402–4. 5780398 10.1128/am.17.3.402-404.1969 PMC377701

[46] Smyser CF Snoeyenbos GH. Evaluation of organic acids and other compounds as Salmonella antagonists in meat and bone meal. Poult Sci. (1979) 58:50–4. 10.3382/ps.0580050 38449

[47] Van Staden JJ Van Der Made HN Jordaan E. The control of bacterial contamination in carcass meal with propionic acid. Onderstepoort J Vet Res. (1980) 47:77–82. 6251412

[48] Ricke SC. Perspectives on the use of organic acids and short chain fatty acids as antimicrobials. Poult Sci. (2003) 82:632–9. 10.1093/ps/82.4.632 12710485

[49] Diener M HelmLe-Kolb C Murer H Scharrer E. Effect of short-chain fatty acids on cell volume and intracellular pH in rat distal colon. Pflügers Arch. (1993) 424:216–23. 8414909 10.1007/BF00384345

[50] Kirchgessner M Roth MX Ergotrope Effekte durch organische Säuren in der Ferkelaufzucht und Schweinemast. [Nutritive effects of organic acids in piglet rearing and pig fattening]. Ubersichten zur Tierernahrung (1988) 16:93–108.

[51] Thompson JL Hinton M. Antibacterial activity of formic and propionic acids in the diet of hens on salmonellas in the crop. B Poult Sci. (1997) 38:59–65. 10.1080/00071669708417941 9088614

[52] Cox NA Davis BH Watts AB Colmer AR. The effect of simulated digestive tract pH levels on survival of three species of Salmonella. Poult Sci. (1972) 51:1268–70. 10.3382/ps.0511268 4567249

[53] Ruhnke I Röhe I Goodarzi Boroojeni F Knorr F Mader A Hafeez A Zentek J. Feed supplemented with organic acids does not affect starch digestibility, nor intestinal absorptive or secretory function in broiler chickens. J Anim Physiol Nutr. (2014) 99(Suppl. 1):29–35. 10.1111/jpn.12313 25865420

[54] Russell JB Another explanation for the toxicity of fermentation acids at low pH: anion accumulation versus uncoupling. J Appl Bacteriol. (1992) 72:363–70.

[55] Fernández-Rubio C Ordó-ez C Abad-González J Barcia-Gallego A Pilar Honrubia M Jose Mallo J. Butyric acid-based feed additives help protect broiler chickens from Salmonella Enteriditis infection. Poult Sci. (2009) 88:943–8. 10.3382/ps.2008-00484 19359681

[56] Hume ME Corrier DE Ivie GW DeLoach JR Metabolism of [14C] propionic acid in broiler chickens. Poult Sci. (1993) 72:786–93. 10.3382/ps.0720786 8502603

[57] Kaczmarek SA Barri A Hejdysz M Rutkowski A. Effect of different doses of coated butyric acid on growth performance and energy utilization in broilers. Poult Sci. (2016) 95:851–9. 10.3382/ps/pev382 26740137

[58] Zhang WH Jiang Y Zhu QF Gao F Dai SF Chen J. Sodium butyrate maintains growth performance by regulating the immune response in broiler chickens. Br Poult Sci. (2011) 52:292–301. 10.1080/00071668.2011.578121 21732874

[59] Gibson GR Roberfroid MB. Dietary modulation of the human colonic microbiota: introducing the concept of prebiotics. J Nutr. (1995) 125:1401–12. 7782892 10.1093/jn/125.6.1401

[60] Durant JA Corrier DE Ricke SC. Short-chain volatile fatty acids modulate the expression of the hilA and invF genes of Salmonella Typhimurium. J Food Prot. (2000) 63:573–8. 10.4315/0362-028X-63.5.573 10826713

[61] Van Loo J Coussement P de Leenheer L Hoebregs H Smits G. On the presence of inulin and oligofructose as natural ingredients in the western diet. Crit Rev Food Sci Nutr. (1995) 35:525–52. 10.1080/10408399509527714 8777017

[62] Wong JM De Souza R Kendall CW Emam A Jenkins DJ. Colonic health: fermentation and short chain fatty acids. J Clin Gastroenterol. (2006) 40:235–43. 10.1097/00004836-200603000-00015 16633129

[63] Tsai CC Hsih HY Chiu HH Lai YY Liu JH Yu B. Antagonistic activity against Salmonella infection *in vitro* and *in vivo* for Lactobacillus strains from swine and poultry. Int J Food Microbiol. (2005) 102:185–94. 10.1016/j.ijfoodmicro.2004.12.014 15992617

[64] Kashket E Bioenergetics of lactic acid bacteria: cytoplasmic pH and osmotolerance. FEMS Microbiol Rev. (1987) 46:233–44.

[65] Chio KH Namkung H Paik IK Effects of dietary fructolligosaccharides on the suppression of intestinal colonization of Salmonella Typhimurium in broiler chickens. Korean J Anim Sci. (1994) 36:271–84.

[66] Gibson GR Wang X Bifidogenic properties of different types of fructooligosaccharides. Food Microbiol. (1994) 11:491–8. 10.1006/fmic.1994.1055

[67] Baurhoo B Letellier A Zhao X Ruiz-Feria CA Cecal populations of Lactobacilli and Bifidobacteria and *Escherichia coli* after *in vivo Escherichia coli* challenge in birds fed diets with purified lignin or mannanoligo-saccharides. Poult Sci. (2007) 86:2509–16. 10.3382/ps.2007-00136 18029796

[68] Biggs P Parsons CM. The effects of Grobiotic-P on growth performance, nutrient digestibilities, and cecal microbial populations in young chicks. Poult Sci. (2008) 87:1796–803. 10.3382/ps.2007-00450 18753447

[69] Lilly DM Stillwell RH. Growth promoting factors produced by probiotics. Science (1965) 147:747–8. 14242024 10.1126/science.147.3659.747

[70] Fuller R Probiotics: their development and use. In: Fuller R Heidt PJ Rusch V van der Waaj, editors. Old Herborn University Seminar Monograph 8. Herborn-Dill: Institute for Microbiology and Biochemistry (1995). p. 1–8.

[71] Havenaar R Huiint Veld JH Probiotics: A general view. In: Wood JB, editor. The Lactic Acid Bacteria. Glasgow: Blackie Academic and Professional (1992). p. 151–70.

[72] Jin L Ho Y Abdullah N Ali M Jalaludin S. Antagonistic effects of intestinal *Lactobacillus* isolates on pathogens of chicken. Lett Appl Microbiol. (1996) 23:67–71. 8987444 10.1111/j.1472-765x.1996.tb00032.x

[73] Van Coillie E Goris J Cleenweck I Grijspeerdt K Botteldoorn N Van Imerseel F. Identification of lactobacilli isolated from the cloaca and vagina of laying hens and characterization for potential use as probiotics to control Salmonella Enteritidis. J Appl Microbiol. (2007) 102:1095–106. 10.1111/j.1365-2672.2006.03164.x 17381753

[74] Hatemink R Non digestible oligosaccharides: healthy food for the colon. In: Proceedings of the International Symposium. Wageningen (1995). p. 1–177.

[75] Gibson GR Hutkins RW Sanders ME Prescott SL Reimer RA Salminen SJ. The International scientific association for probiotics and prebiotics (ISAPP) consensus statement on the definition and scope of prebiotics. Nat Rev Gastroenterol Hepatol. (2017) 14:491–502. 10.1038/nrgastro.2017.75 28611480

[76] Hajati H Rezaei M The application of prebiotics in poultry production. Int J W Poult Sci. (2010) 9:298–304. 10.3923/ijps.2010.298.304

[77] Ricke SC. Potential of fructooligosaccharide prebiotics in alternative and nonconventional poultry production systems. Poult Sci. (2015) 94:1411–8. 10.3382/ps/pev049 25717086

[78] Mundt E Collett SR Berghaus R Pedroso AA Lee MD Maurer JJ. Can bacteriotherapy using commercially available probiotics, prebiotics, and organic acids ameliorate the symptoms associated with runting-stunting syndrome in broiler chickens? Avian Dis. (2015) 59:201–6. 10.1637/122013-Reg 26473669

[79] Hooge DM Meta-analysis of broiler chicken pen trials evaluating dietary mannan oligosaccharide, 1993-2003. Int J Poult Sci. (2004) 3:163–74. 10.3923/ijps.2004.163.174

[80] Kim GB Seo YM Kim CH Paik IK. Effect of dietary prebiotic supplementation on the performance, intestinal microflora, and immune response of broilers. Poult Sci. (2011) 90:75–82. 10.3382/ps.2010-00732 21177446

[81] Geier MS Torok VA Allison GE Ophel-Keller K Hughes RJ Indigestible carbohydrate alter the intestinal microbiota but do not influence the performance of broiler chickens. J Appl Microbiol. (2009) 106:1540–8. 10.1111/j.1365-2672.2008.04116.x 19187131

[82] Midilli M Alp M Kocabagh N Muglah OH Turan N Yilmaz H Çakir S Effects of dietary probiotic and prebiotic supplementation on growth performance and serum IgG concentration of broilers. S Afr J Anim Sci. (2008) 38:21–7. 10.4314/sajas.v38i1.4104

[83] Xu ZR Hu CH Xia MS Zhan XA Wang MQ. Effects of dietary fructooligosaccharide on digestive enzyme activities, intestinal microflora and morphology of male broilers. Poult Sci. (2003) 82:1030–6. 10.1093/ps/82.6.1030 12817461

[84] Farooq U Mohsin M Liu X Zhang H Enhancement of short chain fatty acid production from millet fibres by pure cultures of probiotic fermentation. Trop J Pharm Res. (2013) 12:189–194. 10.4314/tjpr.v12i2.9

[85] Rehman H Hellweg P Taras D Zentek J. Effects of dietary inulin on the intestinal short chain fatty acids and microbial ecology in broiler chickens as revealed by denaturing gradient gel electrophoresis. Poult Sci. (2008) 87:783–9. 10.3382/ps.2007-00271 18340001

[86] Gunal M Yayli G Kaya O Karahan N Sulak O The effects of antibiotic growth promoter, probiotic or organic acid supplementation on performance, intestinal microflora and tissue of broilers. Int J Poult Sci. (2006) 5:149–55. 10.3923/ijps.2006.149.155

[87] Vlademirova L Sourdjiyska S Test on the effect from adding probiotics to the combined feeds for chicks. J Anim Sci. (1996) 3:36–9.

[88] Jin LZ Ho YW Abdullah N Ali MA Jalaluddin S Effects of adherent Lactobacillus cultures on growth, weight of organs and intestinal microflora and volatile fatty acids in broilers. Anim Feed Sci Tec. (1998) 70:197–209.

[89] Vogt H Matthes S Harnisch S Der Einfluss organischer Sauren au fie Leistungen von Broilern und LEgehennen. Archiv fur Geflugelkunde (1981) 45:221–32.

[90] Runho RC Sakomura NK Kuana S Banzatto D Junoqueria OM Stringhini JH Uso do acido organico (acido fumarico) nas racoes de frangos de corte. Brasileira de Zootecnia (1997) 26:1183–1191.

[91] Pascual M Hugas M Badiola JI Monfort JM Garriga M. Lactobacillus salivarius CTC2197 prevents Salmonella Enteritidis colonization in chickens. Appl Environ Microbiol. (1999) 65:4981–6. 10543812 10.1128/aem.65.11.4981-4986.1999 PMC91670

[92] Diez-Gonzalez F Russell JB Factors affecting the extreme acid resistance of Escherichia coli O157:H7. Food Microbiol. (1999) 16:367–74.

[93] Conner D Kotrola J. Growth and survival of Escherichia coli O157:H7 under acidic conditions. Appl Environ Microbiol. (1995) 61:382–5. 7887621 10.1128/aem.61.1.382-385.1995 PMC167295

[94] Kwon YM Ricke SC. Induction of acid resistance of Salmonella Typhimurium by exposure to short-chain fatty acids. Appl Environ Microbiol. (1998) 64:3458–63. 9726897 10.1128/aem.64.9.3458-3463.1998 PMC106747

[95] Kwon YM SY Park SG Birkhold Ricke SC Induction of resistance of Salmonella Typhimurium to environmental stresses by exposure to short-chain fatty acids. J Food Sci. (2000) 65:1037–40. 10.1111/j.1365-2621.2000.tb09413.x

[96] Impey CC Mead GC Fate of salmonellas in the alimentary tract of chickens pre-treated with a mature caecal flora to increase colonization resistance. J Bacteriol. (1989) 66:469–75. 10.1111/j.1365-2672.1989.tb04567.x 2753841

[97] van der Wielen P Biesterveld S Notermans S Hofstra H Urlings BA van Knappen F. Spatial and temporal variation of the intestinal bacterial community in commercially raised broiler chickens during growth. Microb Ecol. (2002) 44:286–29. 10.1007/s00248-002-2015-y 12219265

[98] Gordon HA Bruckner-Kardoss E Staley TE Wagner M Wostmann BS Characteristics of the germfree rat. Acta Anat. (1966) 64:367–389.

[99] Koopman JP Kennis HM Mullink JW Prins RA Stadhouders AM De Boer H. ‘Normalization’ of germfree mice with anaerobically cultured caecal flora of ‘normal' mice. Lab Anim. (1984) 18:188–94. 6379286 10.1258/002367784780891253

[100] Kenworthy R Crabb WE The intestinal flora of young pigs with reference to early weaning and Escherichia coli scours. J Comp Pathol. (1963) 75:215–28. 10.1016/s0368-1742(63)80025-9 14046491

[101] Brisbin JT Gong J Sharif S. Interactions between commensal bacteria and the gut-associated immune system of the chicken. Anim Health Res Rev. (2008) 9:101–10. 10.1017/S146625230800145X 18541076

[102] Oakley BB Buhr RJ Ritz CW Kiepper BH Berrang ME Seal BS. Successional changes in the chicken cecal microbiome during 42 days of growth are independent of organic acid feed additives. BMC Vet Res. (2014) 10:282. 10.1186/s12917-014-0282-8 25427406 PMC4251860

[103] Gilbert ER Williams PM Ray WK Li H Emmerson DA Wong EA. Proteomic evaluation of chicken brush-border membrane during the early posthatch period. J Proteome Res. (2010) 9:4628–39. 10.1021/pr1003533 20687565

[104] Cheled-Shoval SL Amit-Romach E Barbakov M Uni Z. The effect of *in ovo* administration of mannan oligosaccharide on small intestine development during the pre- and posthatch periods in chickens. Poult Sci. (2011) 90:2301–10. 10.3382/ps.2011-01488 21934014

[105] Furuse M Okumura J. Nutritional and physiological characteristics in germ-free chickens. Comp. Biochem Physiol A Physiol. (1994) 109:547–56. 10.1016/0300-9629(94)90193-7 8529001

[106] Gabriel I Lessire M Mallet S Guillot JF Microflora of the digestive tract: critical factors and consequences for poultry. W Poult Sci J. (2006) 62:49–512. 10.1079/WPS2006111

[107] Qu A Brulc JM Wilson MK Law BF Theoret JR Joens LA. Comparative metagenomics reveals host-specific metavirulomes and horizontal gene transfer elements in the chicken cecum microbiome. PLoS ONE (2008) 3:e2945. 10.1371/journal.pone.0002945 18698407 PMC2492807

[108] Saengkerdsub S Anderson RC Wilkinson HH Kim WK Nisbet DJ Ricke SC. Identification and quantification of methangenic archaea in adult chicken ceca. Appl Env Microbiol. (2007) 73:353–6. 10.1128/AEM.01931-06 17085694 PMC1797138

[109] Saengkerdsub S Herrera P Woodward CL Anderson RC Nisbet DJ Ricke SC. Detection of methane and quantification of methanogenic archaea in faeces from young broiler chickens using real-time PCR. Lett Appl Microbiol. (2007) 45:629–34. 10.1111/j.1472-765X.2007.02243.x 17922818

[110] Gong J Forster RJ Yu H Chambers JR Sabour PM Wheatcroft R. Diversity and phylogenetic analysis of bacteria in the mucosa of chicken ceca and comparison with bacteria in the cecal lumen. FEMS Microbiol Lett. (2002) 208:1–7. 10.1111/j.1574-6968.2002.tb11051.x 11934485

[111] Yeoman CJ Chia N Jeraldo P Sipos M Goldenfeld ND White BA. The microbiome of the chicken gastrointestinal tract. Anim Health Res Rev. (2012) 13:89–99. 10.1017/S1466252312000138 22853945

[112] Rehman HU Vahjen W Awad WA Zentek J. Indigenous bacteria and bacterial metabolic products in the gastrointestinal tract of broiler chickens. Arch Anim Nutr. (2007) 61:319–335. 10.1080/17450390701556817 18030916

[113] Danzeisen JL Kim HB Isaacson RE Tu ZJ Johnson TJ. Modulations of the chicken cecal microbiome and metagenome in response to anticoccidial growth promoter treatment. PLoS Biol. (2011) 6:e27949. 10.1371/journal.pone.0027949 22114729 PMC3218064

[114] Gong J Si W Forster RJ Huang R Hai Y Yulong Y Yang C Han Y. 16S rRNA gene-based analysis of mucosa-associated bacterial community and phylogeny in the chicken gastrointestinal tracts: from crops to ceca. FEMS Micro Ecol. (2007) 59:147–57. 10.1111/j.1574-6941.2006.00193.x 17233749

[115] Kohl KD. Diversity and function of the avian gut microbiota. J Comp Physiol B (2012) 182:591–602. 10.1007/s00360-012-0645-z 22246239

[116] Pan D Yu Z. Intestinal microbiome and its interaction with host and diet. Gut Microbes (2013) 5:108–19. 10.4161/gmic.26945 24256702 PMC4049927

[117] Stanley DR Hughes J Moore RJ. Microbiota of the chicken gastrointestinal tract: influence on health, productivity and disease. Appl Microbiol Biotechnol. (2014) 98:4301–10. 10.1007/s00253-014-5646-2 24643736

[118] Waite D Taylor M. Characterising the avian gut microbiota: membership, driving influences and potential function. Front Microbiol. (2014) 5:223. 10.3389/fmicb.2014.00223 24904538 PMC4032936

[119] Dunkley KD Dunkley CS Njongmeta NL Callaway TR Hume ME Kubena LF. Comparison of *in vitro* fermentation and molecular microbial profiles of high-fiber feed substrates incubated with chicken cecal inocula. Poult Sci. (2007) 86:801–10. 10.1093/ps/86.5.801 17435012

[120] Józefiak D Rutkowski A Martin SA Carbohydrate fermentation in the avian ceca: a review. Anim Feed Sci Technol. (2004) 113:1–15. 10.1016/j.anifeedsci.2003.09.007

[121] Hedge SN Rolls BA Coats ME The effects of the gut microflora and dietary fibre on energy utilization by the chick. Br J Nutr. (1982) 48:73–80. 6285954 10.1079/bjn19820089

[122] Oakley BB Lillehoj HS Kogut MH Kim WK Maurer JJ Pedroso A. The chicken gastrointestinal microbiome. FEMS Microbiol Lett. (2014) 360:100–12. 10.1111/1574-6968.12608 25263745

[123] McWhorter TJ Caviedes-Vidal E Karsaov WH. The integration of digestion and osmoregulation in the avian gut. Biol Rev. (2009) 84:533–565. 10.1111/j.1469-185X.2009.00086.x 19673857

[124] Meimandipour A Soleimanifarjam A Azhar K Hair-Bejo M Shuhaimi M Nateghi L Age effects on short chain fatty acids concentrations and pH values in the gastrointestinal tract of broiler chickens. Arch Geflugelkd. (2011) 75:164–8.

[125] Sergeant MJ Constantinidou C Cogan TA Bedford MR Penn CW. Extensive microbial and functional diversity within the chicken cecal microbiome. PLoS ONE (2014) 9:e91941. 10.1371/journal.pone.0091941 24657972 PMC3962364

[126] van der Wielen PW Biesterveld S Notermans S Hofstra H Urlings BA van Knapen F. Role of volatile fatty acids in development of the cecal microflora in broiler chickens during growth. Appl Environ Microbiol. (2000) 66:2536–40. 10.1128/AEM.66.6.2536-2540.2000 10831435 PMC110578

[127] Lu J Idris U Harmon B Hofacre C Maurer JJ Lee MD. Diversity and succession of the intestinal bacterial community of the maturing broiler chicken. Appl Environ Microbiol. (2003) 69:6816–24. 10.1128/AEM.69.11.6816-6824.2003 14602645 PMC262306

[128] Park SH Kim SA Lee SI Rubinelli PM Roto SM Pavlidis HO Original XPCTM effect on Salmonella Typhimurium and cecal microbiota from three different ages of birds when incubated in an anaerobic *in vitro* culture system. Front Microbiol. (2017) 8:1070 10.3389/fmoicb.201701070 28659891 PMC5468444

[129] Park SH Lee SI Christensen K Ricke SC. Comparison of antibiotic supplementation versus a yeast-based prebiotic on the cecal microbiome of commercial broilers. PLoS ONE (2017) 12:e0182805. 10.1371/journal.pone.0182805 28837669 PMC5570483

[130] Kim SA Rubinelli PM Park SH Ricke SC Ability of Arkansas LaKast and LaKast hybrid rice bran to reduce Salmonella Typhimurium in chicken cecal incubations and effects on cecal microbiota. Front Microbiol. (2018) 9:134 10.3389/fmicb.2018.0134

[131] Lee GH Kumar HS Lee JH Chang DH Kim DS Choi SH. Genome sequence of Oscillibacter ruminantium strain GH1, isolated from rumen of Korean native cattle. J Bacteriol. (2012) 194:6362. 10.1128/JB.01677-12 23105088 PMC3486395

[132] Katano Y Fujinami S Kawakoshi A Nakazawa H Oji S Lino T. Complete genome sequence of Oscillibacter valericigenes Sjm18-20 T (=NBRC 101213 T). Stand Genomic Sci. (2012) 6:406–14. 10.4056/sigs.2826118 23408234 PMC3558957

[133] Thibodeau A Fravalo P Yergeau E Arsenault J Lahaye L Letellier A. Chicken caecal microbiome modifications induced by Campylobacter jejuni colonization and by a non-antibiotic feed additive. PLoS ONE (2015) 10:e0131978. 10.1371/journal.pone.0131978 26161743 PMC4498643

[134] Jumpertz R Le DS Turnbaugh PJ Trinidad C Bogardus C Gordon JI. Energy-balance studies reveal associations between gut microbes, caloric load, and nutrient absorption in humans. Am J Clin Nutr. (2011) 94:58–65. 10.3945/ajcn.110.010132 21543530 PMC3127503

[135] DeLange LLM Wijtten PJA Microbial profile in the gastro-intestinal tract of broilers and its relation to feed efficiency. In: Australian Poultry Science Symposium. University of Sydney, Sydney, NSW (2010). p. 3.

